# Total resection of a solitary fibrous tumor of the sellar diaphragm: A case report

**DOI:** 10.3892/ol.2013.1293

**Published:** 2013-04-08

**Authors:** QISHENG ZHONG, SHAOJI YUAN

**Affiliations:** Department of Neurosurgery, General Hospital of Jinan Military Command of Chinese PLA, Jinan, Shandong 250031, P.R. China

**Keywords:** solitary fibrous tumor, total tumor resection

## Abstract

The present study reports the case of a patient with a vision impairment in the right eye. Head computed tomography revealed a round, hyperdense mass in the sellar and suprasellar regions. Pituitary gland magnetic resonance imaging (MRI) revealed isointensity on T1- and T2-weighted imaging. Tumor-enhanced scanning showed heterogeneous contrast enhancement. The initial diagnosis was that of meningioma or pituitary tumor. A total tumor resection was performed using a right pterional approach under general anesthesia. During surgery, the base of the tumor was located on the sellar diaphragm of the left anterior pituitary stalk. The pathological diagnosis was of a solitary fibrous tumor (SFT). The patient had no post-operative diabetes insipidus or idiopathic pituitary hypofunction. The clinical experience, imaging information and pathological features of SFT in this case report may provide a reference for correct diagnosis and total resection of SFTs in the sella turcica.

## Introduction

Solitary fibrous tumor (SFTs) are rare spindle cell tumors arising from the visceral pleura (1) that are rarely located in the central nervous system and even more rarely in the sella turcica. To the best of our knowledge, only 5 cases have been reported in the literature (2–6). SFTs are often undiagnosed due to the use of varied imaging techniques pre-operatively and as they are commonly mistaken for meningiomas or pituitary tumors. Total resection is difficult and complications often develop post-surgery.

The current study presents a rare case of an SFT in the saddle diaphragm, the first in this location. The case report aims to provide information on the clinical experience, imaging and pathological features with regards to SFT, to aid in the correct diagnosis and total resection of tumors in the sella turcica. The case of a patient who presented with a vision impairment in the right eye is discussed. Computed tomography of the head revealed a round, hyperdense mass in the sellar and suprasellar regions. Pituitary gland magnetic resonance imaging (MRI) revealed isointensity on T1 and T2 weighted imaging. Tumor enhanced scanning showed heterogeneous contrast enhancement. The initial diagnosis was that of meningioma or pituitary tumor. A total tumor resection was performed using a right pterional approach under general anesthesia. During surgery, the base of the tumor was located on the sellar diaphragm of the left anterior pituitary stalk. The pathological diagnosis was of a SFT. The patient had no post-operative diabetes insipidus or idiopathic pituitary hypofunction. Written informed consent was obtained from the patient for publication of this case report and any accompanying images

## Case report

**Figure 1:**
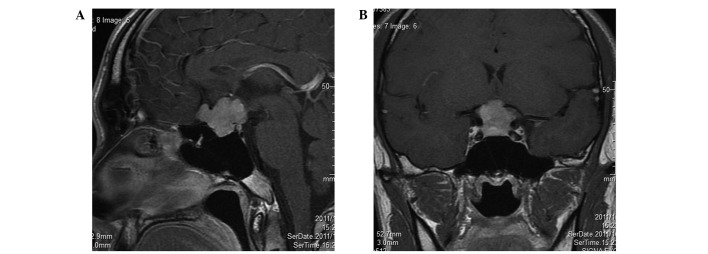
Tumor-enhanced magnetic resonance imaging (MRI) in the pituitary region showing heterogeneous contrast enhancement. (A) Tumor shadow in the sagittal view. (B) Tumor shadow in the coronal view.

A 25-year-old male patient with a 5-month history of aggravated vision through blurring in the right eye was admitted to the General Hospital of Jinan Military Area Command of Chinese PLA, Shandong, China, on October 10th, 2011. An examination revealed sight impairment (vision, right eye, 0.1; left eye, 1.2), a typical temporal hemianopsia of the right eye and locally decreased vision sensitivity of the superior nasal aspect of the left eye, without other positive neurological signs. The serum concentrations of PRL, GH, T3, T4 and TSH were all within normal limits. The patient underwent head computed tomography, which revealed a round, hyperdense mass without clear edges in the sellar and suprasellar regions that was closely associated with the bilateral vessels. Magnetic resonance imaging (MRI) revealed a lobulated, isointense mass wrapped around the anterior communicating artery complex in the sellar-suprasellar region on T1-weighted imaging (T1WI) in the sagittal view. The tumor was isointense and slightly hyperintense in the sellar-suprasellar region in the coronal view. There was no clear border separating the tumor from the peripheral brain tissues. The tumor was also isointense on T2-weighted imaging (T2WI). Tumor-enhanced scanning demonstrated heterogeneous contrast enhancement (Fig. 1). The preliminary diagnosis was of meningioma or pituitary tumor. A tumor resection was conducted using a right pterional approach under general anesthesia on October 15, 2011. Intraoperatively, the base of the tumor was located on the sellar diaphragm of the left anterior pituitary stalk. The tumor pushed the pituitary stalk to the right posterior region, ascended to the suprasellar region, crossed the optic chiasm, invaded the lamina terminalis cistern and wrapped the bilateral A1 segment and anterior communicating artery complex. In the opposite direction, the tumor crossed the saddle-back and reached the slope, showing no clear demarcation from the hypothalamus. Severe adhesion to each side of the optic nerve caused difficulty in the separation of the tissues. The tumor tissue was tough and had an abundant blood supply. A feeding artery was present from a branch of the internal carotid artery in the base of the tumor. A total disparting resection of the tumor was performed with emphasis on left optic neuroprotection under a high-power lens. Following tumor resection, the undamaged pituitary and pituitary stalk were identified; these had been pushed towards the right posterior hypophyseal fossa by the tumor. The patient had no post-operative diabetes insipidus or idiopathic pituitary hypofunction, but had a right eye vision of 0.2, uncorrected and 1.0, corrected. The pathological diagnosis was of a solitary fibrous tumor (SFT).

## Discussion

SFTs are rare spindle cell tumors that arise from the visceral pleura, according to the earliest studies (1). SFTs are rarely located in the central nervous system and even more rarely occur in the sella turcica; to the best of our knowledge, only 5 cases have been reported in the literature. In 2003, Cassarino *et al*(2) first reported an SFT that widely affected the sellar region. A 54-year-old female patient developed an SFT involving the ephippium, sphenoid sinus, internal carotid, interior temporal lobe, ethmoid sinus and pterygoid bone and that extended to the nasopharynx. The tumor was partly removed and the pituitary was found to be undamaged. Kim *et al*(3) described 8 patients with SFTs in the head and neck. Among them, a 56-year-old male patient presenting mainly with visual disturbances was diagnosed as having a pituitary tumor with hemorrhage. The patient underwent partial tumor resection via the transsphenoidal route. A SFT was established by pathology, and a secondary craniotomy was consequently performed. Furlanetto *et al*(4) reported the case of a 28-year-old patient who underwent pituitary adenoma excision via the transsphenoidal approach. The tumor was hard and involved the sellar diaphragm. Complications, including cerebrospinal fluid leakage, idiopathic pituitary hypofunction and meningitis, occurred post-operatively. Yin *et al*(5) reported a 32-year-old male patient with a headache and eye discomfort, who underwent partial pituitary tumor resection and post-operative γ-knife treatment. Cui *et al*(6) described the case of a 29-year-old patient who was pre-operatively diagnosed with a right tuberculum sella meningioma. The pathological diagnosis revealed that the tumor was a malignant SFT. This lesion was pre-operatively diagnosed as a pituitary tumor and meningioma. Prior to surgery, it is almost impossible to achieve a correct diagnosis of SFT in the saddle area. The diagnosis of SFT of the saddle diaphragm in the present case was only achieved by ultimate dependence on the pathological diagnosis, tumor basal position, left anterior displacement of the left optic nerve and the right posterior shift of the pituitary stalk observed in surgery. There have been no previous reports of SFTs of the saddle diaphragm.

However, these tumors have been identified in various locations outside the thoracic cavity, mostly in the extracranial head and neck, including the meninges (7–11), orbit (12), nasal cavity (13), paranasal sinuses (14), soft palate, salivary glands (15) and parapharyngeal space (16). Patients aged 30–64 years are the most commonly affected and there is no gender predilection. The ages of the six patients in the study by Kim *et al*(14) ranged from 46–59 years, with a male to female ratio of 4:2. The 25-year-old male patient in the present study was the youngest among only six known cases of SFT in the saddle area. Visual impairment is a common initial clinical manifestation of SFT caused by the tumor compressing the surrounding tissues, as occurred in the present case. Headaches and hypoglycemia are also common manifestations. However, an endocrine examination disclosed normal pituitary and target gland hormone secretions. In the present case, the SFT had a slightly high and heterogeneous density. The present study agrees with the theory by Kim *et al*(14) that suggests that the density of the tumor correlates with its cellular components. In the present study, the tumor was isointense on the T1- and T2-weighted images. The contrast enhancement was heterogeneous and lower than for meningioma.

**Figure 2:**
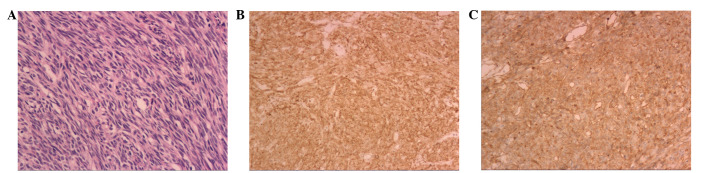
Microscopic examination showing a proliferation of spindle cells with a bundle or flow pattern (hematoxylin and eosin staining). (A) Clusters of spindle cells are readily identifiable. No nuclear atypia, mitotic activity or necrosis are evident (×200). (B) Immunohistochemical examination showing that the tissue is bcl-2-positive (×200). (C) Immunohistochemical examination showing that the tissue is CD34-positive (×100).

The pathological features of SFTs show that the tumors are comprised of spindle or short spindle cells and varying quantities of vascular tissue. Due to the diversity in the number of blood vessels and the uneven cell density, the organizational structure varies; it usually includes alternate distributions of cell-dense and loose areas, and the two areas are often separated by fibers. The tumor cells are spindle or short spindle cells. The tumor tissue may be storiform, bundle-like, fishbone-like, hemangiopericytoma-like (staghorn-like vessels), fence-like or wavy. In the dense areas and porous intervals, collagen fibers with various ranges in thickness and shape are observed. At the endpoint of the disease, the tumor is keloid-like, occasionally with visible radial asbestos-like collagen fibers, and commonly exhibits collagen degeneration of the blood vessel walls. The mesenchyme often shows mucus deterioration and mast cell infiltration with multinucleated cells. Immunohistochemical examination reveals diffuse reactivity for CD34 and vimentin and positive expression for bcl-2 (80–100%) and CD99 (75–100%), but negative expression for EMA and S-100 proteins. This typical presentation of characteristics was evident in the present case; the diffuse reactivity for CD34 and the negative expression of CK, EMA and S-100 protein were observed (Fig. 2). The histological and detailed immunohistological observations confirmed the diagnosis of sellar diaphragm SFT. Cui *et al*(6) stated that atypical and malignant forms of SFT are diagnosed by the following characteristics: increased cell density, apparent nuclear atypia, visible karyokinesis, cell necrosis and resemblance to fibrosarcoma or malignant fibrous histiocytoma. No such characteristics were observed in the present case.

**Figure 3:**
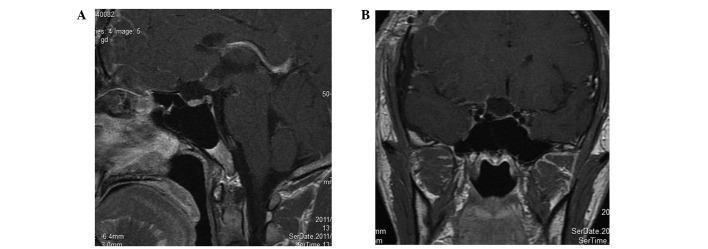
One-week post-operative enhanced magnetic resonance imaging (MRI) demonstrating no tumor recurrence. (A) The tumor shadow has disappeared on the sagittal view. The pituitary and stalk are well preserved. (B) The tumor shadow has disappeared on the coronal view. The pituitary is well preserved.

The majority of SFTs are benign tumors and may be cured following radical surgery. SFT in the sellar region has its own particularity; it is seldomly diagnosed pre-operatively and surgery may be performed by two approaches. If the initial diagnosis is of a pituitary tumor, transsphenoidal surgery may be carried out. This usually causes excessive blood loss and complete removal of the tumor is not possible. Thus, a secondary craniotomy is then required. In the present case, the pre-operative diagnosis was of a pituitary tumor or meningioma, but the two were atypical. To achieve a total resection, surgery was performed via a right pterional approach. Block-cutting resection surgery was carefully conducted through a high-power lens. The tumor base was located on the sellar diaphragm of the left anterior side of the pituitary stalk, where the tumor exhibited apparent bleeding. Following total tumor resection, the anterior communicating artery complex, optic nerve, optic chiasm, pituitary stalk and pituitarium were clearly displayed. A one-week post-operative enhanced MRI revealed no tumor recurrence (Fig. 3).
